# Toripalimab: the First Domestic Anti-Tumor PD-1 Antibody in China

**DOI:** 10.3389/fimmu.2021.730666

**Published:** 2022-01-12

**Authors:** Lin Zhang, Bo Hao, Zhihua Geng, Qing Geng

**Affiliations:** ^1^ Department of Thoracic Surgery, Renmin Hospital of Wuhan University, Wuhan, China; ^2^ Department of Orthopedics of Union Hospital, Tongji Medical College, Huazhong University of Science and Technology, Wuhan, China

**Keywords:** toripalimab, programmed death protein 1, programmed death ligand 1, immunotherapy, tumor, adverse effect

## Abstract

Toripalimab (Tuoyi™) is a selective, recombinant, humanized monoclonal antibody against programmed death protein 1 (PD-1) developed by Shanghai Junshi Bioscience Co., Ltd. Toripalimab is able to bind to PD-1 and block the interaction with its ligands. The binding of toripalimab to PD-1 is mainly attributed to the heavy chain of the former and the FG loop of the latter. Toripalimab received a conditional approval in China for the treatment of melanoma (second-line) in December, 2018. It has also received approvals to treat nasopharyngeal carcinoma (first-line and third-line) and urothelial carcinoma (second-line) in 2021. Additionally, several orphan drug designations were granted to toripalimab by the US Food and Drug Administration. Toripalimab has exhibited primary anti-tumor effects in tumors such as melanoma, lung cancer, digestive tract tumors, hepatobiliary and pancreatic tumors, neuroendocrine neoplasms, nasopharyngeal carcinoma and urothelial carcinoma. It showed a satisfactory anti-tumor effect and long-term survival benefits in Chinese melanoma patients, while the combination of axitinib with toripalimab exhibited an impressive result in metastatic mucosal melanoma. As a checkpoint inhibitor, toripalimab was generally well-tolerated in the enrolled patients. Due to different study populations, comparisons could not be made directly between toripalimab and other drugs in most cases. Nevertheless, the introduction of toripalimab may offer a valuable choice for decision-making in the treatment of tumors in the future.

## Introduction

T cells are important immune cells in the human body and express co-stimulating immune checkpoint proteins on their surface. Through immune checkpoints, cancer cells can block the activation of T cells and their cytotoxic effects on tumors, leading to immune evasion (1). As surface receptor proteins, immune checkpoints can be effectively inhibited by antibodies known as checkpoint inhibitors. The emergence of checkpoint inhibitors has radically changed the landscape of cancer therapy. Among all clinically applied checkpoint inhibitors, anti-programmed death protein 1 (PD-1)/programmed death ligand 1 (PD-L1) antibodies are the most successful (2). Antibody blockade of PD-1 prevents its interaction with PD-L1 and PD-L2, blocking their downstream pathways and recovering the anti-tumor response of T cells (3). There are already well-studied PD-1 inhibitors on the market such as nivolumab and pembrolizumab, two drugs that have been approved by the China National Medical Product Administration (NMPA) and US Food and Drug Administration (FDA) for use in various tumors (4).

Toripalimab (Tuoyi™) is the first domestic anti-PD-1 monoclonal antibody in China with completely independent intellectual property rights (5). Toripalimab has received a conditional approval for the treatment of unresectable or metastatic melanoma that has not responded to previous systemic therapy in December, 2018 (6). Recently, toripalimab was approved by the NMPA for the treatment of recurrent/metastatic nasopharyngeal carcinoma (NPC) and locally advanced or metastatic urothelial carcinoma (UC) (7–9). Showing an acceptable safety profile in clinical studies, it has exhibited promising anti-tumor effects in tumors like melanoma, neuroendocrine neoplasms and urothelial carcinoma, with obvious economic advantages. Here, we summarize the preclinical studies, pharmacological characteristics, anti-tumor effects, and adverse effects (AEs) of this drug in order to provide valuable information for decision-making in the treatment of tumors in the future.

## Preclinical Data and Pharmacological Characteristics of Toripalimab

**Figure 1:**
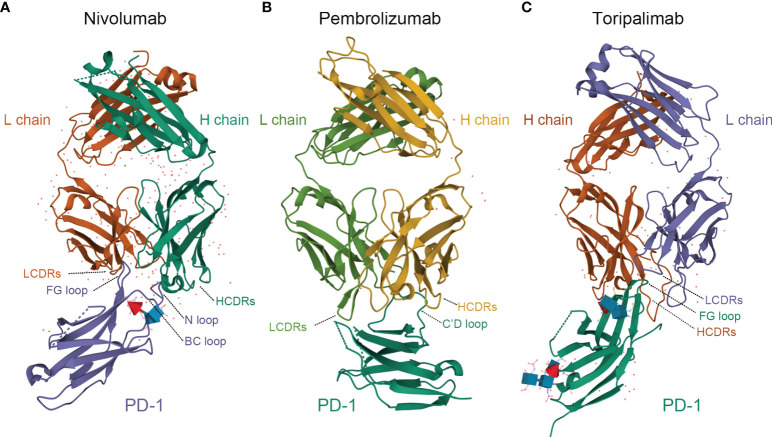
Comparison of structures of PD-1 in complex with nivolumab-Fab **(A)** (PDB code: 5WT9), pembrolizumab-Fab **(B)** (PDB code: 5JXE) and toripalimab-Fab **(C)** (PDB code: 6JBT). L chain, light chain; H chain, heavy chain; LCDRs, complementary determining regions of the light chain; HCDRs, complementary determining regions of the heavy chain; PD-1, programmed death protein 1. Images were acquired from Protein Data Bank and relevant research was conducted by Tan S et al., Na Z et al., and Liu H et al., respectively (12–16).

Toripalimab is the first monoclonal anti-PD-1 antibody approved by the China NMPA onto the market (10). It consists of two heavy chains of 452 amino acids, two light chains of 219 amino acids, and includes an N-linked glycosylation site at N302 in each heavy chain. The average molecular weight of toripalimab is 149,670 Daltons (11–14)(**Figure 1**). Toripalimab is able to bind to PD-1, efficiently blocking the interaction with its ligands; the blockade is mainly attributed to the stereospecific hindrance of the heavy chain (12). The interaction of toripalimab with PD-1 is mainly attributed to the complementarity determining regions of the heavy chain of the former and the FG loop of the latter; the light chain complementarity determining regions of toripalimab participate mainly in recognizing the epitopes on PD-1 (12, 17, 18). Comparatively, nivolumab mainly binds to the N-terminal loop of PD-1, while the binding of pembrolizumab primarily involves the C’ D loop (12, 15, 16) (**Figure 1**).

### Preclinical Safety and Effectiveness

Toripalimab has low effective and inhibitory concentrations. *In vitro*, the effective concentration of toripalimab was determined to be 21 nmol/L and 0.89 ± 0.15 ng/mL by two studies (17, 18). *In vivo*, the effective concentration dose for toripalimab in the MC38 tumor model is between 0.3 and 1 mg/kg (12). In *in vitro* flow cytometry assays, the inhibitory concentrations of toripalimab were 3.0 nmol/L and 3.1 nmol/L for PD-1/PD-L1 and PD-1/PD-L2, respectively (18). In *in vivo* mouse models, by blocking the binding of PD-1 to PD-L1 or PD-L2 using a protein-based enzyme-linked immunosorbent assay, the inhibitory concentrations of toripalimab were determined to be 0.8 nmol/L for PD-L1 and 1.3 nmol/L for PD-L2. Using a cell-based flow cytometry assay, the concentrations were 1.3 nmol/L and 3.7 nmol/L for PD-L1 and PD-L2, respectively (12).

The blockage by toripalimab is effective. *In vivo*, toripalimab can dramatically inhibit the elevated expression of PD-1 on CD4^+^ and CD8^+^ T cells in a dose-dependent manner, while PD-1 receptor occupancy can reach up to 90% at 1 mg/kg and 100% at 10 mg/kg in cynomolgus monkeys (18). In three phase I studies, toripalimab can also bind to the PD-1 receptor on activated T lymphocytes and maintain complete PD-1 receptor occupancy (> 80%) on CD4^+^ and CD8^+^ T cells; this was observed in the majority of the patients in all dose cohorts throughout the observation period (19–21). In mice inoculated with tumor cells, toripalimab can significantly decrease tumor sizes after a 23-day treatment compared with IgG-treated controls (12). Treatment with toripalimab (0.01–10 μg/mL) can also dose-dependently stimulate human T cell proliferation, as well as IFN-γ and TNF-α secretion (18).

### Immunogenicity

Toripalimab provoked only a weak immune response in animal experiments and clinical trials. In an animal experiment, 18 cynomolgus monkeys were treated with toripalimab at low (1 mg/kg), medium (10 mg/kg) and high (75 mg/kg) doses; 10% of monkeys from each group were positive for anti-drug antibodies 28 d after the first administration, indicating a low immunogenicity of this drug (18). In NCT03013101, detection of anti-drug antibodies was performed in 128 melanoma patients treated with toripalimab at 3 mg/kg intravenously once every 2 weeks; samples from 10.2% (13/128) patients were positive after toripalimab treatment and only one positive patient had a decreased toripalimab plasma concentration (22). In NCT02857166, patients were assigned to receive 0.3 mg/kg, 1 mg/kg, 3 mg/kg, 10 mg/kg or 240 mg toripalimab *via* intravenous infusion every 2 weeks; anti-drug antibodies were detected in one (33.3%) patient in the 0.3 mg/kg group, three (42.9%) patients in the 1 mg/kg group, and one (16.7%) patient in the 3 mg/kg group, but were not detected in the 10 mg/kg group or the 240 mg group (19). These results indicate a low immunogenicity of toripalimab in the body.

### Pharmacological Characteristics

In the phase II study POLARIS-01, when toripalimab was given at 3 mg/kg per 2 weeks (Q2W), its steady-state median trough plasma concentration was 39.8 μg/mL (22). In phase I studies, the serum concentrations of toripalimab reached a maximum at about 1 h, within 2 h and within 6 h after infusion, respectively (19–21). In NCT02836795 and NCT02836834, the steady state trough concentration of toripalimab was 8.9 ± 4.4, 37.8 ± 17.5, 174.3 ± 95.6 μg/mL and 8.5 ± 2.6, 34.5 ± 10.0, and 195.8 ± 104.6 μg/mL for the 1 mg/kg, 3 mg/kg, and 10 mg/kg cohorts, respectively (20, 21). In NCT02857166, the analyses of blood samples of patients receiving toripalimab showed a serum half-life of 150–222 h after a single infusion and 188–525 h after multi-dose infusions (19). In another two studies, the average serum half-lives for the 1, 3 and 10 mg/kg cohorts were 6.2, 7.7, 9.8 days and 7.7, 8.0 and 14.2 days respectively after a single infusion; after multi-dose infusions, the half-lives were 9.5, 16.5, 13.9 days and 10.7, 12.1 20.1 days, respectively (20, 21). Based on the preclinical and pharmacological data, toripalimab was tested in therapeutic clinical trials.

## Efficacy of Toripalimab in Tumors

**Figure 2:**
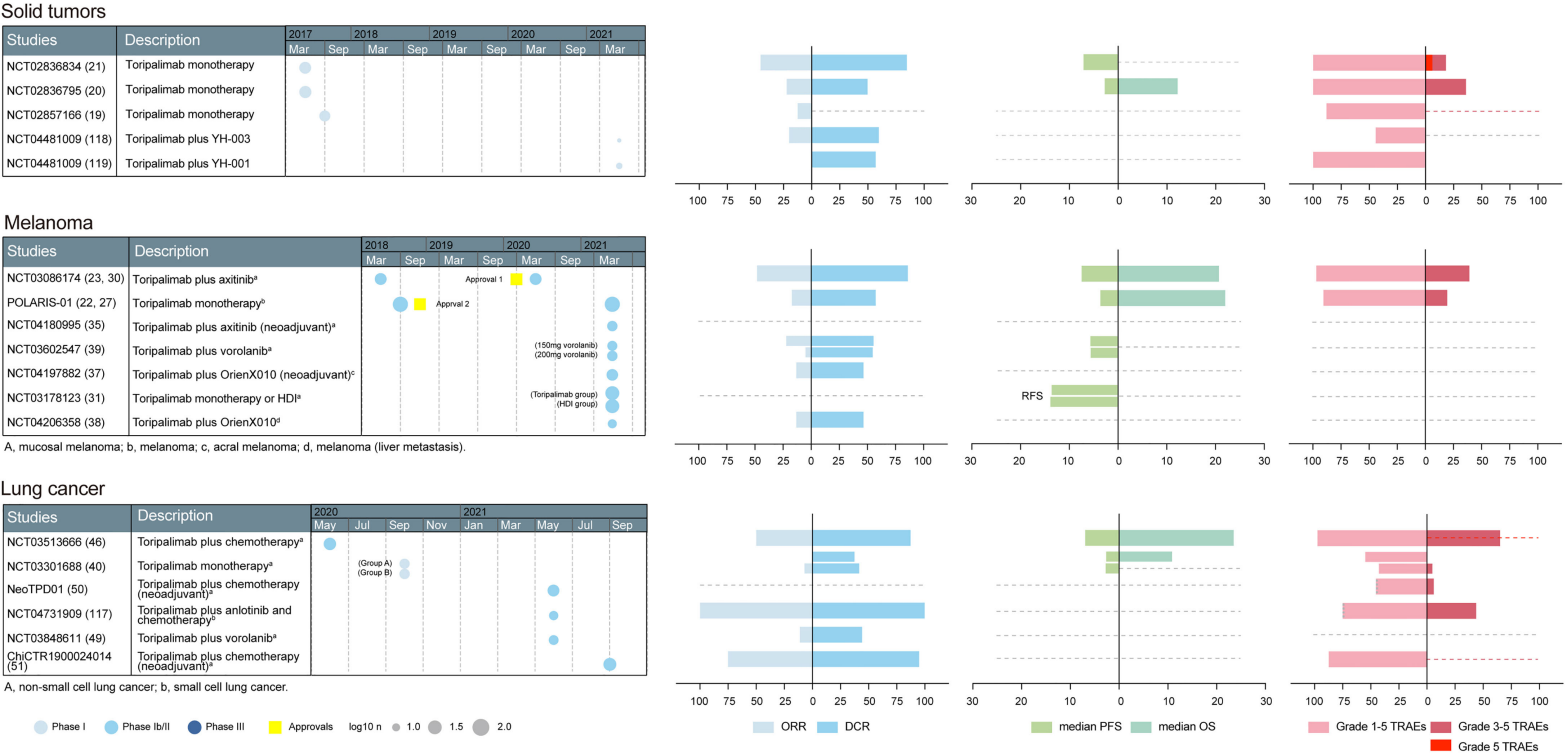
A summary of prospective clinical studies of toripalimab on solid tumors, melanoma and lung cancer. Dates indicate the time when the results of the studies were available online. Dashed lines perpendicular to the y-axis in the bar graph indicate absence of corresponding available data. Dotted gray line at the edge of column means not less than the indicated number. Approval 1, approval in mucosal melanoma, an orphan designation by the FDA (plus axitinib); approval 2, approval in melanoma, by the NMPA (second-line); HDI, high-dose IFN-α2b; ORR, overall response rate; DCR, disease control rate; RFS, recurrence-free survival; PFS, progression-free survival; OS, overall survival; TRAEs, treatment-related adverse events; FDA, Food and Drug Administration; NMPA, National Medical Products Administration.

**Figure 3:**
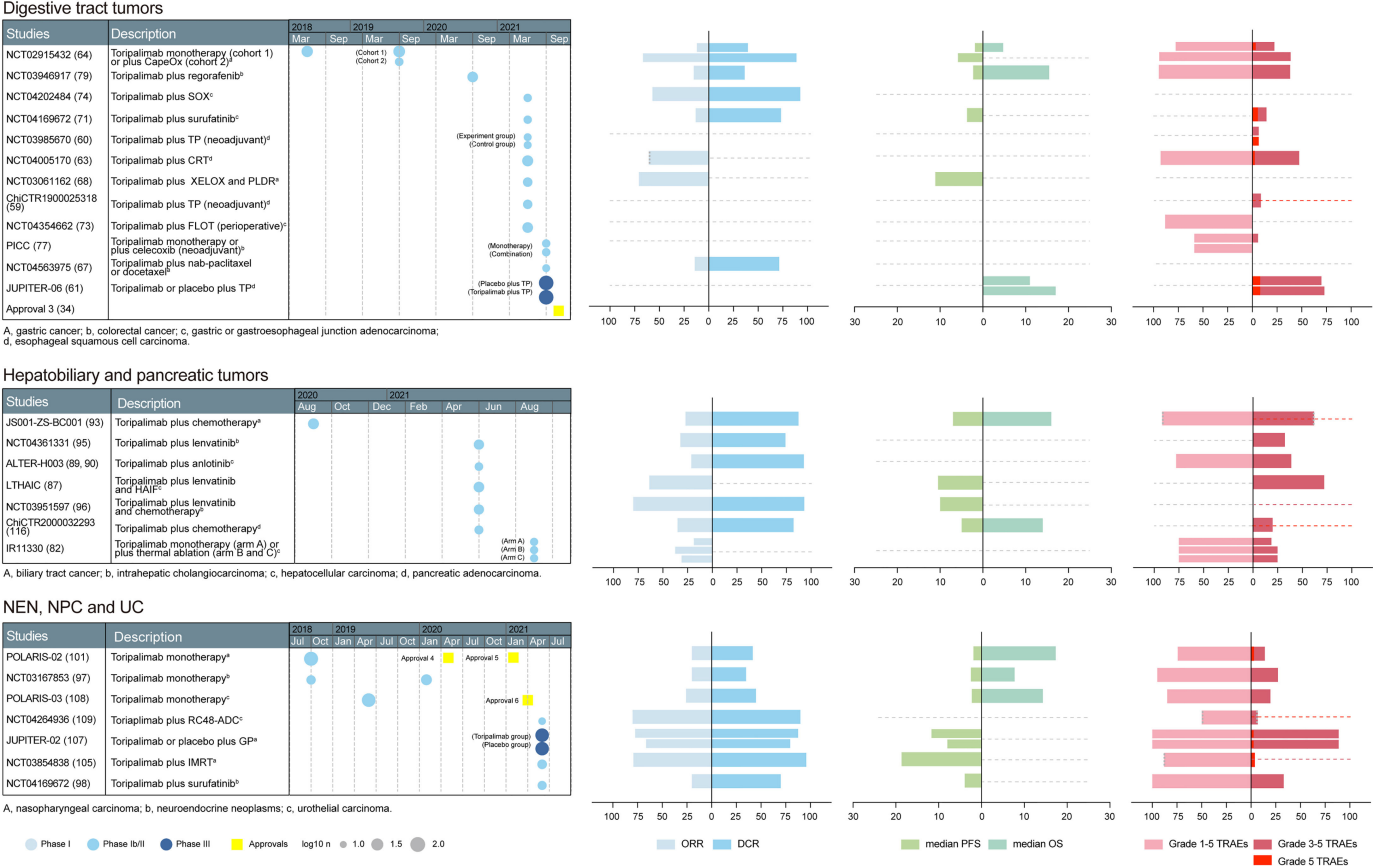
A summary of prospective clinical studies of toripalimab on digestive tract tumors, hepatobiliary and pancreatic tumors, and other tumors. Dates indicate the time when the results of the studies were available online. Dashed lines perpendicular to the y-axis in the bar graph indicate absence of corresponding available data. Dotted gray line at the edge of column means not less than the indicated number. CapeOX, capecitabine and oxaliplatin; SOX, S-1 plus oxaliplatin; TP, paclitaxel plus platinum; CRT, chemoradiotherapy; XELOX, capecitabine and oxaliplatin; PLDR, pulsed low dose rate radiotherapy; FLOT, 5-fluorouracil, leucovorin, oxaliplatin, docetaxel; HAIF,hepatic arterial infusion of oxaliplatin, 5-fluorouracil, and leucovorin; GP, gemcitabine and platinum; IMRT, intensity-modulated radiotherapy; approval 3, approval in esophageal cancer, an orphan designation by the FDA; approval 4, approval in NPC, an orphan designation by the FDA; approval 5, approval in NPC, by the NMPA (third-line); approval 6, approval in UC, by the NMPA (second-line); ORR, overall response rate; DCR, disease control rate; RFS, recurrence-free survival; PFS, progression-free survival; OS, overall survival; TRAEs, treatment-related adverse events; FDA, Food and Drug Administration; NMPA, National Medical Products Administration.

Based on the above preclinical studies, toripalimab was tested in phase I clinical studies. The recommended phase II dose was determined to be 3 mg/kg Q2W in a first-in-human phase I trial (NCT02836795) (23). In NCT02836795, patients in the dose escalation cohorts received intravenous infusion of toripalimab at 1 mg/kg, 3 mg/kg, and 10 mg/kg Q2W; 28 days after the first dose, subjects continued to receive toripalimab at the intended dose level Q2W. The confirmed dosage of toripalimab laid the foundation for further clinical trials. The primary endpoints are usually safety and tolerability in phase I studies; overall response rate (ORR), defined as the percentage of patients achieving complete response (CR) or partial response (PR), in phase II studies; and overall survival (OS) and progression-free survival (PFS) in phase III studies (24). The basic information of toripalimab studies in these cancers was visualized in **Figures 2**, **3**.

### Efficacy of Toripalimab in Melanoma

Melanoma of the skin accounts for 1.7% of newly-diagnosed cancers and 0.6% of cancer-related deaths worldwide (25).Clinical trials in melanoma have largely been unsuccessful, and there are few treatment options when the disease becomes advanced (26). The first approval of toripalimab was for unresectable or metastatic melanoma that has failed to respond to previous systemic therapy, which was based on a phase II study (NCT03013101/POLARIS-01) (22). Additionally, the anti-tumor effects of nivolumab and pembrolizumab were investigated in NCT00730639 and KEYNOTE-151, respectively. The information of toripalimab studies in melanoma was visualized in **Figure 2** and the comparisons between toripalimab, nivolumab and pembrolizumab in melanoma are listed in **Table 1**.

**Table 1:** Comparison of different therapeutic regimens for melanoma.

Therapeutic Regimen		Toripalimab[NCT03013101/POLARIS-01 [(22, 27)]	Nivolumab[NCT00730639 (28)]	Pembrolizumab[KEYNOTE-151 (29)]	Toripalimab plus axitinib[NCT03086174 (23, 30)]
Patient races		Chinese	Not mentioned	Chinese	Chinese
ORR	total	17.3% (22/127)	30.8% (33/107)	16.7% (17/102)	NA
	cutaneous	20.3% (16/79)	NA	17.7% (14/79)	NA
	mucosal	0 (0/22)	NA	13.3% (2/15)	48.3% (14/29)
DCR	total	57.5% (73/127)	37.4% (40/107)	38.2% (39/102)	NA
	cutaneous	57.0% (45/79)	NA	42.1% (16/38) for acral	NA
	mucosal	40.9% (9/22)	NA	20.0% (3/15)	86.2% (25/29)
Median PFS	total	3.6 months	3.7 months	2.8 months	NA
	cutaneous	3.2 months for acral, 5.5 months for NC	NA	NA	NA
	mucosal	1.9 months	NA	NA	7.5 months
Median OS	total	22.0 months	16.8 months	12.1 months	NA
	cutaneous	16.9 months for acral, 46.1 months for NC	NA	NA	NA
	mucosal	10.3 months	NA	NA	20.7 months
Safety profiles	Grade 1-5 TRAEs	90.6% (116/128)	54.2% (58/107)	84.5% (87/103)	97.0% (32/33)
	Grade ≥3 TRAEs	19.5% (25/128)	22.4% (24/107)	10.7% (11/103)	39.4% (13/33)
	Grade 5 TRAEs	0	0	1.9% (2/103)	0

ORR, overall response rate; DCR, disease control rate; PFS, progression-free survival; OS, overall survival; NA, not available/applicable; NC, nonacral cutaneous; TRAE, treatment-related adverse event.

Overall, the toripalimab was effective in melanoma. In POLARIS-01, toripalimab was given at 3 mg/kg Q2W until disease progression or unacceptable toxicity in 128 Chinese patients who had previously received systemic therapy. The achieved ORR was 17.3% (22/127), and the disease control rate (DCR), defined as the percentage of patients achieving CR, PR, or stable disease (SD), was 57.5% (73/127); the median PFS and OS were 3.6 months and 22.2 months, respectively (22). Toripalimab has led to long-term survival benefit, and PD-L1 positive patients show markedly longer OS than PD-L1 negative patients; the data for different subtypes are shown in **Table 1** (22, 27). In 22 melanoma patients from another phase I study (NCT02836795), ORR, DCR, PFS and OS were 18.2% (4/22), 45.5% (10/22), 84 days and 448 days, respectively (20). Comparatively, in KEYNOTE-151, pembrolizumab was administered in previously-treated Chinese melanoma patients; the achieved ORR was 16.7% (17/102) and the DCR was 38.2% (39/102). The ORRs were 15.8% (6/38) for acral, 19.5%(8/41) for non-acral and 13.3% (2/15) for mucosal melanoma patients, the DCRs were 42.1% (16/38) for acral melanoma and 20.0% (3/15) for mucosal melanoma, respectively (29). In NCT00730639, overall response and disease control were achieved by nivolumab in 30.8% (33/107) and 37.4% (40/107) patients, respectively; the median PFS and median OS were 3.7 months and 16.8 months (28). The ORR achieved by nivolumab appears to be the highest among all three drugs; however, the efficacies of nivolumab for each subtype are unknown, and the study population in NCT00730639 was not mentioned. In addition, adjuvant toripalimab was able to improve the recurrence-free survival of mucosal melanoma patients, but its comparison with conventional chemotherapy remained to be conducted (31).

The combination of toripalimab and axitinib, a vascular endothelial growth factor inhibitor, has shown a potent effect in treating mucosal melanoma. Vascular endothelial growth factor is an important growth factor for the growth of cancer and is upregulated in melanoma patients (32). Simultaneous blockade of PD-1 and vascular endothelial growth factor receptor 2 can induce a synergistic anti-tumor effect in mice inoculated with colon cancer cells (33). In a phase Ib study (NCT03086174), 33 Chinese patients were given axitinib 5 mg twice a day plus toripalimab 1 or 3 mg/kg every 2 weeks until confirmed disease progression, unacceptable toxicity, or voluntary withdrawal, in a dose-escalation and a cohort-expansion phase. The achieved ORR was 48.3% (14/29) and DCR was 86.2% (25/29), while the median PFS was 7.5 months (23). The combinatorial plan produced a high response rate. Thus, an orphan designation for treatment of mucosal melanoma was granted to the combinational therapy by the US FDA in March 25, 2020 (34). An updated analysis of NCT03086174 revealed a median OS of 20.7 months among these patients (30). Later, toripalimab plus axitinib combination treatment was tested in an adjuvant setting, and led to pathological complete response (pCR), defined as no viable tumor on all slides, in 14.3% (2/14) of patients (35). In addition, the effectiveness of the combination in advanced mucosal melanoma is currently being investigated in a phase II trial (registration number: NCT03941795) (36).

Toripalimab has also been tested for the treatment of melanoma in other settings. For example, the pCR achieved by toripalimab plus OrienX010, a granulocyte-macrophage colony-stimulating factor, is 14.3% (3/21) in resectable stage IIIb–IVM1a acral melanoma patients; and intravenous administration of toripalimab plus intralesional injection of OrienX010 achieved an ORR of 13.3% (2/15) in liver metastasis (37, 38). Toripalimab plus vorolanib achieved a 13.2% (5/38) ORR in advanced mucosal melanoma as a first-line therapy (39). Additionally, a randomized, multi-center, phase II study (NCT03430297) comparing toripalimab as a first-line treatment of metastatic melanoma to dacarbazine (a first-line chemotherapy for melanoma) is currently ongoing (36). The results of these studies may provide more indications for toripalimab in the future.

### Efficacy of Toripalimab in Non-Small Cell Lung Cancer

Toripalimab exhibited anti-tumor effects in non-small cell lung cancer (NSCLC) as a single agent (**Figure 2**). In a phase I study (NCT02836834), the ORR and DCR achieved by toripalimab in seven Chinese NSCLC patients were 14.3% (1/7) and 71.4% (5/7), respectively (21). In another phase I study (NCT03301688), 41 heavily-treated patients were included and treated with a single dose of toripalimab; the ORR and DCR achieved were 7.1% (2/28) and 39.3% (11/28), respectively (40). The effect of toripalimab in NSCLC was also observed in retrospective studies (41). In comparison, nivolumab and pembrolizumab monotherapy achieved ORRs of 17.1% (22/129) and 19.4% (96/495) and DCRs of 27.1% (35/129) and 41.2% (204/495) in NSCLC, as revealed by the phase I studies CHECKMATE-003 and KEYNOTE-001, respectively (42, 43). Due to the small sample size and different patient baseline characteristics such as race of NCT02836834, it is not valid to compare the effect of toripalimab with the other two drugs directly.

Toripalimab plus other therapies has also exerted anti-tumor effects in NSCLC. Multiple target cytotoxic T-lymphocyte cells can restore antitumor immunity to improve patient outcome. In NCT04193098, a combination of multiple target cytotoxic T-lymphocyte cells and toripalimab as a second-line therapy led to an ORR of 38.4% (5/13) and a DCR 76.9% (10/13) in patients with advanced NSCLC, without causing treatment-related death (44). Platinum-based chemotherapy is one of the standard systemic therapies for NSCLC (45). In NCT03513666, 40 Chinese NSCLC patients who failed to respond to first-line EGFR-TKIs and did not harbor T790M mutation were enrolled and received toripalimab combined with carboplatin and pemetrexed every 3 weeks, for up to six cycles, followed by toripalimab and pemetrexed maintenance until disease progression or unacceptable toxicity. The ORR achieved by the combination regime was 50.0% (20/40), the median PFS was 7.0 months and the median OS was 23.5 months, better than historical data for traditional chemotherapy (46). The ORR and DCR achieved by toripalimab combined with chemotherapy in a retrospective study were 39.2% and 96.2% among 79 NSCLC patients (47). In addition, a phase III clinical trial comparing the toripalimab versus placebo in combination with chemotherapy in the same setting as NCT03924050 is ongoing (48). Also, toripalimab in combination with vorolanib, a multi-target tyrosine kinase inhibitor, also exhibited anti-tumor effects as a second-line therapy (49).

A toripalimab-containing regimen in a neoadjuvant setting yielded a high major pathological response (MPR) rate and caused no treatment-related surgical delays. In a phase 2 study (NeoTPD01/NCT04304248), surgically resectable stage IIIA or T3–4N2 IIIB NSCLC patients received three cycles of neoadjuvant treatment with intravenous toripalimab plus carboplatin, and pemetrexed or nab-paclitaxel (50). MPR, defined as less than 10% residual tumor remaining at the time of surgery, was achieved in 66.7% (20/30) of patients, and pCR was achieved in 50.0% (15/30) of patients. Treatment discontinuation or dose reduction was not observed and there were no treatment-related deaths (50). In another study, neoadjuvant toripalimab plus chemotherapy led to a lower pCR rate (18.2%) and MPR rate (40.9%), but resulted in an ORR of 75.0% (30/40) and DCR of 95.0% (38/40) (51). Nivolumab and pembrolizumab monotherapy have also been tested in a neoadjuvant setting in US medical centers and an Israeli medical center; the MPRs achieved were 42.9% (9/21) and 40% (4/10), respectively (52–54). In contrast, Sheng et al. tested pembrolizumab in a Chinese setting; they enrolled 37 Chinese adults with untreated, surgically resectable stage IIB–IIIB squamous NSCLC and treated them with two cycles of pembrolizumab with albumin-bound paclitaxel plus carboplatin (55). All tumors were completely resected and MPR occurred in 24 (64.9%) resected tumors; none of the patients discontinued neoadjuvant therapy due to toxic effects and no treatment-related death was observed (55).

In addition, ongoing trials conferred potential possibilities in NSCLC treatment. For example, in NCT04238169, metastatic NSCLC patients who have previously received first-line platinum-based chemotherapy or immune checkpoint inhibitors (except toripalimab) and diagnosed with confirmed disease progression were included and treated with stereotactic body radiation therapy (30–50 Gy/5 F for 2–4 lesions) plus toripalimab (240 mg, Q3W) with or without bevacizumab (7.5 mg/kg, Q3W) until disease progression or intolerable toxicity (56). Toripalimab was also tested in different NSCLC stages in prospective studies, such as in early-stage NSCLC, and treatment-naive advanced NSCLC (57). Release of these results in the future may lay the foundations for more indications.

### Efficacy of Toripalimab in Esophageal Cancer

Toripalimab plus chemotherapy is effective when used as a neoadjuvant therapy in esophageal squamous cell carcinoma (ESCC) (**Figure 3**). Combinational fluoropyrimidine plus platinum-based chemotherapy is a recommended first-line treatment for advanced or metastatic ESCC, but confers limited survival benefits (58). In a phase II study (ChiCTR1900025318), 23 subjects with resectable ESCC were included and given toripalimab combined with paclitaxel and cisplatin as neoadjuvant treatment. Among the evaluable patients, 33.3% (6/18) achieved pCR (59). The combination regime also showed a controllable safety profile, and grade 3–4 treatment-related adverse events (TRAEs) were reported in two patients. Interestingly, when toripalimab was administrated two days after chemotherapy rather than being used simultaneously, the combinational neoadjuvant regime showed a trend toward a higher pCR rate (36.4%, 4/11 versus 7.7%, 1/13, P = 0.079) (60).

Toripalimab plus chemotherapy conferred better OS and PFS than chemotherapy alone in the first-line setting (**Figure 3**). In the randomized, double-blind phase III study JUPITER-06, 514 Chinese patients with treatment-naive, advanced or metastatic ESCC were randomized (1:1) to receive 240 mg toripalimab or placebo in combination with paclitaxel plus cisplatin every 3 weeks for up to six cycles, followed by toripalimab or placebo maintenance (61). Incidence of fatal TRAEs was similar between the groups (8.2% vs 8.2%), while significant advantages in OS and PFS for toripalimab over placebo (HR = 0.58, HR = 0.58) were observed, with median OS being 17.0 months and 11.0 months, respectively (61). In a similar setting (KEYNOTE-590), pembrolizumab plus 5-fluorouracil and cisplatin (n = 373) also led to superior OS (12.6 months versus 9.8 months, HR = 0.72) and PFS (6.3 months versus 5.8 months, HR = 0.65) to placebo plus 5-fluorouracil and cisplatin (n = 376) in patients with advanced ESCC from 168 medical centers in 26 countries, with a lower TRAE-related death rate (7.5%, 28/373; 10.1%, 38/376) (62).

Efficacy of toripalimab was also observed in other combinatorial regimes (**Figure 3**). For example, when combined with concurrent chemoradiotherapy, toripalimab achieved a clinical complete response rate of 60.7% (17/28) in previously untreated ESCC patients at 3 months (63). Based on the satisfactory anti-tumor effects of toripalimab shown above, an orphan designation was designated by FDA on November 8, 2021 (34).

### Efficacy of Toripalimab in Gastric and Gastroesophageal Junction Cancer

The efficacy of toripalimab in advanced gastric cancer (GC) has been observed and seems promising (**Figure 3**). In the phase Ib part of the clinical trial NCT02915432, 58 Chinese chemo-refractory advanced GC patients received toripalimab (3 mg/kg d1, Q2W) as a monotherapy plan, which resulted in an ORR of 12.1% (7/58) (64). In cohort 1 of KEYNOTE-059, 259 patients from different countries with advanced GC received pembrolizumab monotherapy; the ORR in the east Asia subgroup was 8.8% (3/34) (65). In the gastric cohort of KEYNOTE-012, pembrolizumab monotherapy was associated with an ORR of 22.2% (8/36) in patients from different countries/regions with PD-L1-positive recurrent or metastatic adenocarcinoma of the stomach or gastro-esophageal junction (66). The east Asian data of KEYNOTE-012 are unavailable. In contrast, KEYNOTE-059 is more comparable to NCT02915432.

The combination of toripalimab and chemotherapy was effective in advanced GC patients (**Figure 3**). For example, the toripalimab plus CapeOx (capecitabine and oxaliplatin) regime (NCT02915432) as a first-line treatment in chemotherapy-naive advanced GC patients achieved an ORR and DCR of 66.7% (12/18) and 88.9% (16/18), respectively (64). In patients with advanced gastric adenocarcinoma, toripalimab plus nab-paclitaxel/docetaxel as second-line treatment resulted in confirmed PR in 1/7 patients, and SD in 4/7 patients, corresponding to an ORR of 14.2% and a DCR of 71.4% (67). The toripalimab plus XELOX (capecitabine and oxaliplatin) chemotherapy regimen and pulsed low dose rate radiotherapy also showed therapeutic effects in abdominal metastasis of advanced GC, with an ORR of 70.8% (17/24) (68). In ATTRACTION-4, nivolumab in combination with CapeOx yielded an ORR and DCR of 76.5% (13/17) and 88.2% (15/17), respectively, in treatment-naive advanced GC patients from Japan and South Korea (69). In cohort 2 of KEYNOTE-059, previously untreated advanced GC patients received pembrolizumab, cisplatin, and 5-fluorouracil (or capecitabine in Japan); the combination plan yielded an ORR of 60.0% (15/25) and a DCR of 80.0% (20/25) (70). The data of NCT02915432 and ATTRACTION-4 are more comparable because their study populations are similar.

Toripalimab showed an anti-tumor effect in gastric or gastroesophageal junction adenocarcinoma (G/GEJ) in different settings (**Figure 3**). Surufatinib is a novel small-molecule kinase inhibitor targeting VEGFRs, FGFR and CSF-1R, and its combination with toripalimab resulted in two confirmed PR and six SD in 15 evaluable G/GEJ patients who did not respond to first-line systemic chemotherapy, with median PFS being 3.71 months (71). FLOT (docetaxel, oxaliplatin, leucovorin, 5-FU) is the standard perioperative treatment for resectable G/GEJ (72). The combination of toripalimab and FLOT produced a 25.0% pCR and 42.9% MPR among 28 G/GEJ patients receiving complete resection (73). A toripalimab plus S-1 and oxaliplatin regimen as a first-line treatment showed effectiveness (ORR 57.1%, and DCR 92.8%) among 14 patients with advanced stage G/GEJ (74).

### Efficacy of Toripalimab in Colorectal Cancer

Toripalimab alone or plus celecoxib conferred therapeutic effects in colorectal cancer (CRC) patients in a neoadjuvant setting (**Figure 3**). Cancer cell-intrinsic cyclooxygenases-2 expression contributed to resistance to immune checkpoint blockade (75). Therapeutically targeting the cyclooxygenases-2 pathway with widely used non-steroidal and steroidal anti-inflammatory drugs was able to synergize with immune checkpoint blockers and strengthen their anti-tumor effects in mouse CRC models (76). Celecoxib is a non-steroidal anti-inflammatory drug. In the phase 2 PICC study, toripalimab with or without celecoxib was tested in a neoadjuvant setting. In PICC, 34 patients with histologically-confirmed mismatch repair-deficient or microsatellite instability-high CRC, with clinical stage T3–T4 or any T with lymph node positivity were included and randomly assigned (1:1) to receive either toripalimab plus celecoxib (combination group) or toripalimab monotherapy (monotherapy group). While neither treatment caused surgical delays, pCR was achieved in 88.2% (15/17) in the combination group and 64.7% patients in the monotherapy group (77).

Toripalimab plus regorafenib showed anti-tumor efficacy in metastatic CRC (**Figure 3**). Regorafenib is an oral tyrosine kinase inhibitor targeting angiogenesis, the tumor microenvironment and tumor immunity, and has been approved for the treatment of metastatic CRC after failing standard therapies (78). In the phase I/II study NCT03946917, regorafenib plus toripalimab was tested in relapsed or metastatic CRC patients that had failed ≥ 2 previous lines of chemotherapy or were intolerant to prior systemic treatment (79). Twelve patients were enrolled in the dose escalation phase, which identified a dose of 80 mg regorafenib plus 3 mg/kg toripalimab to be the recommended phase II dose. In the 33 evaluable patients treated with recommended phase II doses, the ORR was 15.2% (5/33), the DCR was 36.4% (12/33), the median PFS was 2.1 months and median OS was 15.5% at the data cutoff of July 12, 2020 (79). In a retrospective analysis, the ORR in these patients is 12.1% (4/33), DCR is 36.4% (12/33), and median PFS is 3.8 months (80). In a similar setting to NCT03946917, nivolumab achieved an ORR of 36.0% (9/25) in the Japanese population (81).

### Efficacy of Toripalimab in Hepatobiliary Cancers

Toripalimab alone or in combination with other treatments showed promising anti-tumor effects in advanced hepatocellular carcinoma (HCC) (**Figure 3**). Toripalimab monotherapy led to an ORR of 18.8% in previously-treated advanced HCC patients (82). Additional ablation increased the response rate in these patients, without causing treatment-related death (82, 83). Lenvatinib has been reported to improve the survival of advanced HCC patients, while hepatic arterial infusion of oxaliplatin, 5-fluorouracil, and leucovorin (HAIF) led to further survival benefit (84, 85). In a retrospective analysis, the addition of toripalimab plus HAIF prolonged PFS and OS and increased ORR in HCC patients receiving lenvatinib (86). The effect of the combination regime was also explored in a prospective phase II trial, showing an ORR of 63.9% and median PFS of 10.5 months among 36 treatment-naive advanced HCC patients (87). Anlotinib, a novel multi-targeting tyrosine kinase inhibitor, has shown promising efficacy and safety as a first- or second-line treatment strategy in advanced HCC (88). The combination of toripalimab and anlotinib conferred an ORR of 21.4% (3/14) and a DCR of 92.9% (13/14) among treatment-naive patients with advanced HCC (89, 90).

Toripalimab plus chemotherapy showed a preliminary efficacy in treatment-naive advanced biliary tract cancer (BTC) (**Figure 3**). Combination chemotherapy with gemcitabine plus cisplatin has been regarded as the standard treatment for patients with advanced BTC, but confers only limited clinical benefit (91). In the phase II trial NCT03796429, treatment-naive patients with advanced BTC received toripalimab combined with gemcitabine and S-1 until progressive disease or unacceptable toxicity. By the data cutoff at January 24, 2021, the ORR was 27.1% (13/48) and DCR was 87.5% (42/48), median PFS was 7.0 months and median OS was 16.0 (92, 93). In a similar phase I study JapicCTI-153098, 30 chemotherapy-naive Japanese patients with unresectable or recurrent BTC were given nivolumab and cisplatin plus gemcitabine chemotherapy; in this cohort, median OS was 15.4 months, median PFS was 4.2 months, and 11 of 30 patients (36.7%) had an objective response (94).

Toripalimab plus lenvatinib showed promising efficacies in intrahepatic cholangiocarcinoma (ICC) (**Figure 3**). In NCT04361331, 31 pathologically-confirmed advanced ICC patients were included and treated with toripalimab plus lenvatinib as a first-line therapy. The combination plan resulted in an ORR of 32.3% (10/31) and a DCR of 74.2% (23/31) (95). When the regime was added to oxaliplatin and gemcitabine chemotherapy, a standard chemotherapy regime for ICC, the ORR became 80% (24/30) and DCR 93.3% (28/30) in treatment-naive patients (96). Treatment-related death was identified in neither of the studies, which indicated an acceptable safety profile.

### Efficacy of Toripalimab in Neuroendocrine Neoplasms

Toripalimab has a satisfactory efficacy when used alone or in combination with surufatinib in neuroendocrine neoplasms (NEN) (**Figure 3**). The ORR achieved in previously-treated metastatic Chinese NEN patients was 20.0% (8/40), and DCR was 35.0% (14/40) (NCT02939651) (97). Furthermore, four patients achieved confirmed PR and 10 achieved SD among 20 tumor evaluable patients receiving toripalimab plus surufatinib, with a median PFS of 3.94 months and no treatment-related deaths (98). In contrast, pembrolizumab monotherapy resulted in ORR and DCR of 3.4% (1/29) and 24.1% (7/29), respectively, in grade 3 NEN patients refractory to first-line chemotherapy (NCT02939651) (99). It has been widely acknowledged that the prognosis of NEN based on pathological grading is dependent on the measurement of a proliferative index such as Ki-67 (100). In NCT02939651, patients had Ki-67 > 20%, while in NCT03167853, patients had Ki-67 ≥ 10%. Thus, the higher ORR achieved by toripalimab might be correlated with the lower grade of tumors in NCT03167853 to some extent. In addition, the comparison should still be made cautiously because the patient races in the two studies are different.

### Efficacy of Toripalimab in Nasopharyngeal Cancer

Toripalimab was shown to be effective when used alone or with radiotherapy in treating NPC patients (**Figure 3**). In POLARIS-02/NCT02915432, 190 treated Chinese patients with recurrent or metastatic NPC were included and received 3 mg/kg toripalimab Q2W *via* intravenous infusion until disease progression, unacceptable toxicity, or voluntary withdrawal (101). Toripalimab monotherapy led to an ORR of 20.5% (39/190) and a DCR of 41.6% (79/190), which led to its approval for treating patients with recurrent/metastatic NPC as a third-line therapy (8). Due to the satisfactory anti-tumor effect of toripalimab shown in NPC, an orphan designation was also given by the FDA in May 18, 2020 (34). In comparison, the ORRs achieved by nivolumab in NCI-9742/NCT02339558 and pembrolizumab in KEYNOTE-028/NCT02054806 were 20.5% (9/44) and 26.3% (5/19), respectively (102, 103). Patients in these studies have similar tumor stages and treatment status. In NCI-9742, 82.2% (37/45) of patients are Asian; in KEYNOTE-028, this number is 63.0% (17/27). Considering the small sample size and lower proportions of Asian patients in KEYNOTE-028, the comparison between NCT02915432 and NCI-9742 is more valid. Intensity-modulated radiotherapy is the most widely used radiotherapy technique for NPC and significantly improves patient survival (104). In a phase II trial in patients with recurrent NPC, toripalimab in combination with intensity-modulated radiotherapy was prescribed, achieving an ORR of 79.2% (19/24) and a DCR of 95.8% (23/24), causing one treatment-related death (4.0%) (105).

Toripalimab plus chemotherapy showed superior clinical benefit compared to chemotherapy alone in NPC patients (**Figure 3**). Gemcitabine-cisplatin (GP) was the standard first-line chemotherapy regime for advanced or metastatic NPC (106). Recently, the phase III study JUPITOR-02 was published, making a head-to-head comparison between toripalimab plus GP and placebo plus GP (107). The study involved 289 patients with recurrent or metastatic NPC and no previous chemotherapy history. The patients were randomized (1:1) to receive toripalimab plus GP (combination group) or placebo plus GP (placebo group); longer PFS was observed in the combination group compared to the placebo group (11.7 versus 8.0 months); the data for median OS were not available yet, but a trend toward better OS was observed, with the stratified HR for OS being 0.78 (107). Due to the superior clinical benefit conferred by the combination plan, NMPA have approved the indication of toripalimab plus GP as a first-line therapy in NPC recently (7).

### Efficacy of Toripalimab in Urothelial Carcinoma

Toripalimab produced a satisfactory anti-tumor effect in locally advanced or metastatic UC patients (**Figure 3**). In a phase I study NCT02836795, toripalimab monotherapy achieved an ORR of 25.0% (2/8) and a DCR of 67.5% (5/8) among eight Chinese patients with metastatic UC (20). In the phase II study POLARIS-03/NCT03113266, 151 Chinese patients with advanced metastatic UC that had failed prior standard therapy were enrolled and treated with toripalimab until disease progression, unacceptable toxicity or voluntary withdrawal (108). By the cut-off date, there were two CR, 37 PR, and 29 SD, corresponding to an ORR of 25.8% (39/151) and a DCR of 45.0% (68/151) (65). When combined with RC48-ADC, a novel humanized anti-HER2 antibody-drug conjugate, the ORR increased to 80% (8/10) and DCR to 90% (9/10) in patients that were mostly treatment-naive (8/14) (109). The satisfactory anti-tumor effect of toripalimab shown in UC facilitated toripalimab’s approval in previously-treated locally advanced or metastatic UC (9). In CHECKMATE-275, nivolumab 3 mg/kg Q2W resulted in an ORR of 19.6% (52/265) in locally advanced or metastatic UC patients (110), and in KEYNOTE-052, pembrolizumab in treatment-naive patients yielded an ORR of 24.1% (89/370) (111). Although the patients had received prior systemic therapy, the response rate in POLARIS-03 is still higher than in KEYNOTE-052. However, it should be noted that study populations of CHECKMATE-275 and KEYNOTE-052 are different from that of POLARIS-03.

### Efficacy of Toripalimab in Other Cancers

In addition to the above-mentioned tumors, toripalimab monotherapy has also shown anti-tumor effects in phase I studies on alveolar soft part sarcoma, lymphoma and renal cell carcinoma, achieving ORRs of 25.0% (3/12), 90.9% (10/11) and 33.3% (2/6), respectively (20, 21). In lymphoma patients, the ORR achieved by toripalimab (NCT02836834) is apparently higher than those achieved by nivolumab [CHECKMATE-205, 69.1% (168/243)] or pembrolizumab [KEYNOTE-087, 69.0% (145/210)]. Although the sample size in NCT02836834 is small and the patient race is different from the other two studies, the result suggests a promising anti-tumor effect of toripalimab in lymphoma that is worth exploring in future studies (21, 112, 113). As neoadjuvant therapies, toripalimab-containing regimes were claimed to be effective in penile squamous cell carcinoma and anal canal squamous carcinoma (114, 115). Toripalimab was also effective in cancers such as pancreatic adenocarcinoma and small-cell lung cancer (**Figures 2**, **3**) (116, 117). It is interesting that the regime achieved a 100% ORR in extensive-stage small cell lung cancer (117). In addition, toripalimab in combination with YH-001, an anti-CTLA-4 monoclonal antibody, or YH-003, an anti-CD40 monoclonal antibody, were shown to be effective in phase I studies in advanced solid tumors (**Figure 2**) (118, 119). In other retrospective studies, toripalimab was claimed to be effective when combined with other treatments in patients with soft tissue sarcoma cervical cancer, and head and neck squamous cell carcinoma (120–122). These studies have indicated a bright future for this drug in clinical applications. The efficacies of toripalimab in different clinical studies have been visualized in **Figures 2**, **3**.

## Adverse Effects

Although toripalimab has shown preliminary efficacy in the treatment of various tumors, there are inevitable side effects. The adverse effects have five grades, which were defined according to the Common Terminology Criteria for Adverse Events from the National Cancer Institute, referring to mild, moderate, severe and life-threatening AEs or death, in ascending order, and were coded using the Medical Dictionary for Regulatory Activities (123, 124).

Generally, the safety profile of toripalimab is acceptable; the incidence of AEs is higher than well-studied checkpoint inhibitors, but is manageable. The incidences of all grade TRAEs have been visualized in **Figures 2**, **3**, and most common TRAEs are summarized in **Table 2**. The safety of checkpoint inhibitors has been assessed in previous research, revealing an incidence varying between 54% and 76% for all TRAEs (125). The incidences of fatal AEs for PD-1 inhibitors and PD-L1 inhibitors were shown to be 0.361% (33/9136) and 0.63% (12/3164) in a study that comprehensively evaluated the spectrum of fatal checkpoint inhibitor-related toxic effects (126). For toripalimab, there was no dose-limiting toxicity in three phase I studies, and the pooled incidence rates of AEs and AE-related death (hereafter “the rates”) were 96.8% (91/94) and 2.1% (2/94) (19–21), respectively. The death rate appears to be higher than the average; however, the sample sizes are small, meaning that it is difficult to draw a conclusion. In the first reported phase I study of nivolumab, the rates were 69.9% (207/296) and 1.0% (3/296) (127). The rates for pembrolizumab in a phase I study were 70.9% (351/495) and 0.2% (1/495) (43). When toripalimab was used alone, TRAEs occurred in 42.9–100% patients across the studies, and the death rate ranged from 0–6.7%. In combination regimes, the highest incidence of grade 5 TRAEs was 8.2%, when combined with chemotherapy (**Figure 3**). The incidences may not be compared directly because the baseline characteristics of patients are different.

**Table 2:** Summary of most common treatment-related adverse events of all grades across 15 prospective studies with complete results.

Trials	Tumor	Patient number	Included TRAE	Regimen	Leukopenia	Anemia	Neutropenia	Nausea	Fatigue	ALT elevation	AST elevation	Decreased appetite/Anorexia	Thrombocytopenia	Rash/Skin reaction	Vomiting	Hypothyroidism	Fever	Constipation	Diarrhea	Creatine kinase elevation	Pruritus	Hyponatremia	Hyperglycemia	Myalgia	Proteinuria	Peripheral neuropathy
NCT03946917 (79)	CRC	39	All	Toripalimab plus regorafenib	0.0%	5.1%	2.6%	0.0%	10.3%	0.0%	0.0%	5.1%	10.3%	30.8%	0.0%	2.6%	20.5%	0.0%	17.9%	0.0%	2.6%	0.0%	0.0%	12.8%	2.6%	0.0%
PICC (77) (Combination)	CRC	17	All	Toripalimab plus celecoxib (neoadjuvant)	0.0%	0.0%	0.0%	6.0%	12.0%	6.0%	18.0%	0.0%	0.0%	6.0%	0.0%	6.0%	0.0%	0.0%	0.0%	0.0%	12.0%	0.0%	0.0%	0.0%	0.0%	0.0%
PICC (77) (Monotherapy)	CRC	17	All	Toripalimab monotherapy (neoadjuvant)	0.0%	0.0%	0.0%	18.0%	24.0%	6.0%	12.0%	6.0%	0.0%	18.0%	0.0%	0.0%	6.0%	0.0%	0.0%	0.0%	18.0%	0.0%	0.0%	0.0%	0.0%	0.0%
NCT02915432 (64) (Cohort 1)	GC	58	All (>5%)	Toripalimab monotherapy	8.6%	12.1%	1.7%	5.2%	10.3%	8.6%	10.3%	5.2%	6.9%	8.6%	5.2%	12.1%	5.2%	0.0%	5.2%	0.0%	10.3%	0.0%	0.0%	0.0%	8.6%	0.0%
NCT02915432 (64) (Cohort 2)	GC	18	All (>10%)	Toripalimab plus CapeOx	38.9%	27.8%	38.9%	50.0%	16.7%	16.7%	33.3%	27.8%	22.2%	22.2%	38.9%	0.0%	11.1%	16.7%	27.8%	0.0%	22.2%	0.0%	0.0%	0.0%	22.2%	0.0%
NCT03013101 (22)	Melanoma	128	All (>5%)	Toripalimab montherapy	20.3%	0.0%	17.2%	0.0%	14.8%	31.3%	22.7%	10.2%	7.0%	23.4%	0.0%	27.3%	6.3%	0.0%	0.0%	25.8%	0.0%	0.0%	30.5%	5.5%	0.0%	0.0%
NCT03086174 (23)	Mucosal melanoma	33	All (>15%)	Toripalimab plus axitinib	27.3%	21.2%	24.2%	21.2%	48.5%	42.4%	33.3%	24.2%	0.0%	36.4%	0.0%	51.5%	0.0%	0.0%	60.6%	36.4%	0.0%	0.0%	33.3%	0.0%	57.6%	0.0%
NCT03167853 (97)	NEN	40	All (≥10%)	Toripalimab monotherapy	15.0%	22.5%	10.0%	15.0%	20.0%	32.5%	37.5%	10.0%	0.0%	17.5%	0.0%	0.0%	12.5%	0.0%	12.5%	25.0%	25.0%	15.0%	35.0%	0.0%	40.0%	0.0%
POLARIS-02 (101)	NPC	190	All (≥5%)	Toripalimab monotherapy	10.0%	15.3%	5.3%	0.0%	13.2%	13.7%	15.3%	0.0%	0.0%	6.3%	0.0%	23.7%	9.5%	0.0%	0.0%	0.0%	8.4%	0.0%	0.0%	0.0%	12.6%	0.0%
NCT03854838 (105)	NPC	25	All	Toripalimab plus IMRT	16.0%	0.0%	8.0%	76.0%	88.0%	20.0%	12.0%	0.0%	4.0%	40.0%	8.0%	32.0%	4.0%	0.0%	4.0%	20.0%	32.0%	0.0%	36.0%	16.0%	0.0%	0.0%
JUPITER-02 (107) (Toripalimab group)	NPC	146	All (≥10%)	Toripalimab plus GP	91.1%	88.4%	85.6%	69.2%	35.6%	36.3%	37.7%	53.4%	63.0%	27.4%	67.1%	30.8%	30.8%	39.0%	30.1%	17.8%	16.4%	25.3%	0.0%	23.3%	0.0%	30.1%
JUPITER-02 (107) (Placebo group)	NPC	143	All (≥10%)	Placebo plus GP	94.4%	94.4%	93.0%	83.2%	35.7%	39.9%	30.8%	58.7%	62.2%	21.7%	65.7%	16.8%	21.7%	44.8%	23.1%	23.8%	7.0%	36.4%	0.0%	26.6%	0.0%	28.7%
NCT03301688 (40)	NSCLC	41	All	Toripalimab monotherapy	2.4%	0.0%	0.0%	4.9%	2.4%	7.3%	12.2%	0.0%	0.0%	14.6%	0.0%	7.3%	0.0%	0.0%	0.0%	0.0%	2.4%	0.0%	0.0%	0.0%	0.0%	0.0%
NeoTPD01 (50)	NSCLC	33	All	Toripalimab plus chemotherapy (neoadjuvant)	0.0%	51.6%	6.1%	30.3%	15.2%	0.0%	0.0%	12.1%	15.2%	18.2%	3.0%	18.2%	0.0%	0.0%	3.0%	0.0%	3.0%	0.0%	0.0%	15.1%	0.0%	15.1%
NCT03513666 (46)	NSCLC	40	All	Toripalimab plus chemotherapy	82.5%	67.5%	70.0%	47.5%	25.0%	50.0%	52.5%	37.5%	47.5%	15.0%	17.5%	0.0%	10.0%	27.5%	0.0%	0.0%	0.0%	0.0%	0.0%	0.0%	0.0%	0.0%
NCT02857166 (19)	ST	25	All	Toripalimab monotherapy	4.0%	0.0%	0.0%	8.0%	64.0%	4.0%	4.0%	16.0%	0.0%	24.0%	0.0%	12.0%	4.0%	0.0%	12.0%	0.0%	12.0%	0.0%	0.0%	0.0%	16.0%	0.0%
NCT02836834 (21)	ST	33	All (≥10%)	Toripalimab monotherapy	30.0%	18.0%	18.0%	0.0%	18.0%	18.0%	21.0%	0.0%	0.0%	21.0%	0.0%	0.0%	30.0%	0.0%	0.0%	3.0%	15.0%	0.0%	0.0%	0.0%	0.0%	0.0%
NCT02836795 (20)	ST	36	All (≥20%)	Toripalimab monotherapy	25.0%	33.3%	0.0%	0.0%	22.2%	22.2%	25.0%	22.2%	0.0%	44.4%	0.0%	0.0%	38.9%	0.0%	0.0%	0.0%	25.0%	0.0%	58.3%	0.0%	50.0%	0.0%

CRC, colorectal cancer; GC, gastric cancer; NEN, neuroendocrine neoplasms; NPC, nasopharyngeal carcinoma; NSCLC, non-small cell lung cancer; ST, solid tumors; TRAE, treatment-related adverse events; CapeOx, capecitabine and oxaliplatin; IMRT, intensity-modulated radiotherapy; GP, gemcitabine and platinum; ALT, alanine aminotransferase; AST, aspartate aminotransferase.

Toripalimab has shown a better safety profile than pembrolizumab in melanoma. In POLARIS-01, toripalimab monotherapy resulted in 90.6% (116/128) TRAEs and 19.5% (25/128) grade 3 or 4 TRAEs, with no TRAE-related deaths, in melanoma patients (22). The rates for combination of toripalimab with axitinib were 97.0% (32/33) and 0% (23). For pembrolizumab monotherapy in melanoma, 84.5% (87/103) patients encountered TRAEs, and 8.7% (9/103) patients encountered grade 3 or 4 TRAEs; 1.9% (2/103) of patients died (one patient died of shock and one died of pulmonary embolism), although the researchers claimed that the deaths were unrelated to treatment (29). In a pooled analysis, nivolumab monotherapy caused 66.3% (57/86), 76.4% (508/665) TRAEs and 8.1% (7/86), 12.5% (83/665) grade 3 or 4 TRAEs in mucosal and cutaneous melanoma patients, without deaths. The pooled incidence of TRAEs and grade 3 or 4 TRAEs were 75.2% (565/751) and 12.0% (90/751), respectively (128). However, the analysis included patients of multiple races, compared to only Chinese patients in POLARIS-01 and KEYNOTE-151.

In other tumors, toripalimab also exhibited manageable adverse effects. Chinese patients may react differently from other populations; thus, the rates of toripalimab and the other two drugs may not be compared directly. The TRAE incidence of toripalimab in prospective clinical studies were shown and compared in **Figures 2**, **3**. The safety profile of toripalimab could be analyzed systematically in future research.

## Discussion

Toripalimab is the first domestic PD-1 antibody in China and has received approvals for the treatment of advanced melanoma, NPC and UC (128). The binding of toripalimab to PD-1 is mainly attributed to the heavy chain of the former and the FG loop of the latter. Toripalimab has shown preliminary anti-tumor effects comparable to pembrolizumab or nivolumab in tumors like melanoma, GC, NEN and UC, with acceptable safety profiles. Prospective clinical studies on toripalimab were shown in integrated tables and graphs in **Figures 2**, **3**.

There are some disadvantages and limitations of this review. Firstly, all patients included in toripalimab studies are native Chinese, which makes it difficult to compare the anti-tumor effects with other drugs. Future studies involving other races may provide more comparable data. Secondly, although the comparisons made between studies are based on their identical primary endpoints, there is a lack of direct head-to-head comparisons. The comparisons between studies need to be made prudently, considering the different baseline characteristics of patients. Importantly, there are mainly phase 2 studies for toripalimab at present. Comparisons between drugs could be made using the indirect comparison method, which requires phase 3 randomized controlled studies with control groups (129–131). Furthermore, complete data from some studies, such as JUPITER-06 and ChiCTR1900025318, are not available. The release of complete study results will solve this problem.

Despite these limitations, there are some inherent advantages of this study. Firstly, there are more than 200 ongoing clinical trials of toripalimab, with future results expected (36). Furthermore, toripalimab has a lower cost than the other two well-studied PD-1 inhibitors (nivolumab and pembrolizumab) after the patient-assistant program; after the negotiation of Chinese government with pharmaceutical companies and the inclusion of toripalimab into the medical insurance catalogue, the cost of toripalimab further decreased (132–134). The approval of toripalimab has provided tumor patients with an extra option. Despite the decline in cost of the domestic PD-1 antibody in China, the radical change in the drug price may also exert pressure on foreign pharmaceutical companies and lead to a price decline in their PD-1/PD-L1 antibodies. Moreover, several orphan drug designations have been granted to toripalimab by the US FDA (34). Thus, it may also benefit tumor patients in other countries.

## Conclusion

In conclusion, toripalimab has shown preliminary efficacy in tumors like melanoma, lung cancer, ESCC, GC, G/GEJ, CRC, HCC, BTC, ICC, NEN, NPC, and UC, with acceptable safety profiles. Specifically, it has a potent efficacy in melanoma according to data from relevant studies. Although there are some limitations, it is hopeful that the introduction of toripalimab will provide a valuable option for tumor patients. Meanwhile, our review has also provided oncologists with information on a potential choice for the treatment of cancers in the future.

## Author Contributions

Conception and design: LZ. Manuscript writing: LZ and BH. Revision and language editing: LZ, ZG. Supervision: QG. All authors contributed to the article and approved the submitted version

## Conflict of Interest

The authors declare that the research was conducted in the absence of any commercial or financial relationships that could be construed as a potential conflict of interest.

## Publisher’s Note

All claims expressed in this article are solely those of the authors and do not necessarily represent those of their affiliated organizations, or those of the publisher, the editors and the reviewers. Any product that may be evaluated in this article, or claim that may be made by its manufacturer, is not guaranteed or endorsed by the publisher.
